# A Randomized Study of the Effect of Replacing Sugar-Sweetened Soda by Reduced Fat Milk on Cardiometabolic Health in Male Adolescent Soda Drinkers

**DOI:** 10.3390/nu12020405

**Published:** 2020-02-04

**Authors:** Sally Chiu, Patty Siri-Tarino, Nathalie Bergeron, Jung H. Suh, Ronald M. Krauss

**Affiliations:** 1 Children’s Hospital Oakland Research Institute; 5700 Martin Luther King Jr. Way, Oakland, CA 94609, USA; schiu@chori.org (S.C.); pattysiri@gmail.com (P.S.-T.); nbergeron@chori.org (N.B.); suh@dnli.com (J.H.S.); 2 Department of Pediatrics, University of California, San Francisco, San Francisco, CA 94143, USA; 3 Siri Tarino Consulting, Piedmont, CA 94611, USA; 4 Department of Biological and Pharmaceutical Sciences, College of Pharmacy, Touro University California, Vallejo, CA 94592, USA

**Keywords:** obesity, sugar-sweetened beverage, soda, milk, lipid, lipoprotein, blood pressure

## Abstract

Soda consumption in adolescents has been linked to poorer metabolic outcomes. We tested whether replacing soda with reduced fat milk would improve features of atherogenic dyslipidemia and other cardiometabolic risk factors. Thirty overweight and obese adolescent boys who were habitual consumers of sugar-sweetened beverages were randomly assigned to consume 24 oz/day of sugar-sweetened soda or an energy equivalent of reduced fat (2%) milk for 3 weeks with crossover to the alternate beverage after a ≥ 2 weeks washout. Plasma lipids and lipoproteins and other laboratory measures were assessed after each beverage period. Lipid and lipoprotein measurements, C-reactive protein, and serum transaminases did not differ significantly between the soda and milk phases of the study. Systolic blood pressure *z*-score and uric acid concentration were significantly lower after consuming milk compared to soda. Milk consumption also significantly decreased plasma glucosyl ceramide (d18:1/C16:0) and lactosylceramides (d18:1/C16:0 and d18:1/C18:0). While no effects of replacing soda with milk on lipid and lipoprotein measurements were observed in these normolipidemic weight-stable adolescent boys, decreases in systolic blood pressure, uric acid, and glycosphingolipids suggest that an overall favorable effect on cardiometabolic risk can be achieved following a short-term dietary intervention.

## 1. Introduction

Sugar-sweetened beverages (SSBs) are a major source of energy intake among adolescents, averaging approximately 9–10% of total daily kilocalories for children 12–19 years [1]. Historically, the increase in SSB consumption in the United States has paralleled the rise in rates of obesity [2] and related co-morbidities, including atherogenic dyslipidemia, hypertension, and insulin resistance in both adults [3,4] and children [5,6], suggesting that SSBs are detrimental to cardiometabolic health. Although SSB consumption in adolescents has declined slightly in recent years, absolute levels of intake remain high [1]. Furthermore, milk consumption among adolescents, which has declined in recent decades, remains below the recommended daily intake [1], an issue of particular concern given the nutrients that milk provides, including calcium, magnesium, potassium, and vitamin D. Notably, substitution models have indicated that replacing sugar-sweetened beverages with milk is inversely associated with weight gain in children and adolescents [7].

Clinical trials have demonstrated deleterious effects of SSBs as well as fructose on lipid and lipoprotein profiles relative to milk and other beverages [8,9,10]. A study in free living adults showed that after 6 months, 32 ounces per day of SSBs (~100 g sucrose) resulted in increased fasting plasma triglyceride and total cholesterol levels compared to an equivalent amount of milk, water, or diet beverages [10]. Another study in adults randomly assigned to six different beverage interventions providing varying sources and amounts of sugars (fructose, glucose, sucrose) showed that daily consumption of SSB with 80 g sucrose or 80 g fructose for 3 weeks resulted in no significant differences in traditional lipid parameters, including plasma total, low-density lipoprotein (LDL), and high-density lipoprotein (HDL) cholesterol, and triglycerides [9], but there was a decrease in LDL peak particle size due to a shift in the distribution of LDL from larger to smaller species that are associated to a greater extent with cardiovascular disease risk [11]. In a cross-sectional study in children, total fructose was found to be the only dietary predictor of LDL particle size [12], and in children with obesity and metabolic syndrome, fructose restriction in exchange for complex carbohydrates resulted in increased LDL size [13]. Other cardiometabolic parameters, including blood pressure, fat volume, and inflammatory markers, have been shown to improve with replacement of SSBs with milk and other drinks, such as water and non-caloric diet drinks in adults [9,10]. These data are consistent with findings from the Harvard Pooling Project of Diet and Coronary Disease, which suggest a decrease in risk of coronary events when milk is substituted for SSBs [14]. It is not known, however, whether replacement of SSBs with milk improves markers of cardiometabolic risk in adolescents in a similar fashion. 

The objective of the present study was to test whether replacement of 24 ounces of sugar-sweetened soda per day (~80 g sugar/day) with an energy equivalent amount of reduced fat (2%) milk would increase LDL peak particle diameter in a cohort of overweight adolescent boys who were habitual soda drinkers. A two-period randomized crossover study was implemented with evaluation of lipids and lipoproteins, measures of adiposity, and blood pressure after each 3-week intervention period during which soda or milk was consumed on a daily basis in conjunction with the participants’ usual diets. Exploratory outcomes were insulin resistance as assessed by homeostatic model assessment of insulin resistance (HOMA-IR) and inflammation as assessed by C-reactive protein (CRP), as well as serum uric acid and serum transaminases. In addition, measurements were made of a panel of plasma sphingolipids (SPL), including ceramides (Cer), sphingoid bases (SB), and sphingomyelins (SM), that have been linked to a variety of metabolic diseases and inflammatory conditions [15,16,17].

## 2. Materials and Methods

### 2.1. Study Design

**Figure 1:**
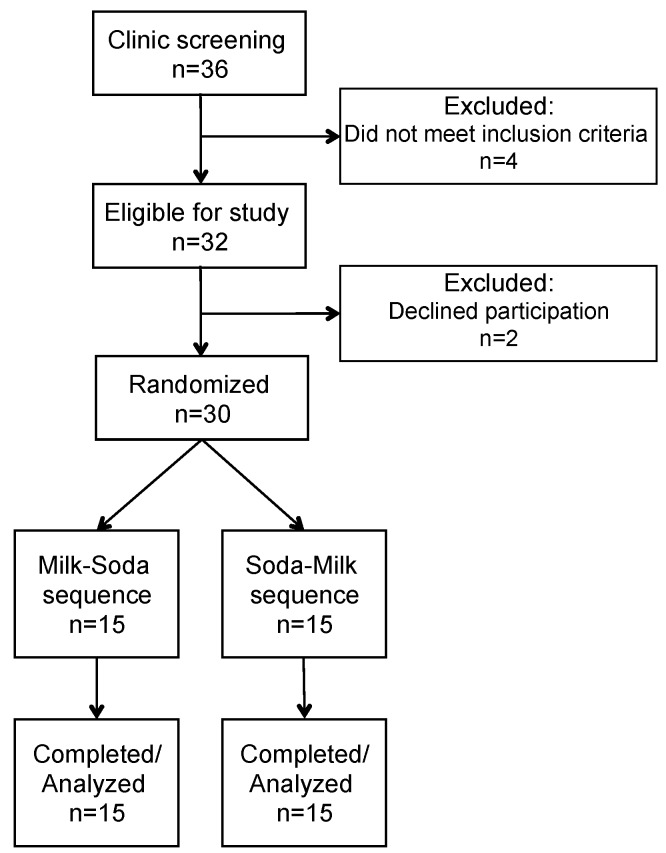
Consolidated Standards of Reporting Trials (CONSORT) flow diagram.

This was a two-period randomized study in 30 overweight or obese adolescent males who were self-reported habitual consumers of SSBs conducted at the Cholesterol Research Center (Berkeley, CA, USA) from June 2014 to September 2016. As assigned beverages added to their habitual diet, participants consumed soda (24 oz per day; sweetened with high fructose corn syrup) or an energy equivalent amount of 2% milk (22 oz per day) in random order for 3 weeks each, separated by a ≥2-week washout period (Figure 1). Previous studies have shown that a three-week dietary intervention is sufficient to induce changes in plasma lipids and lipoproteins [18,19] and changes in response to high fructose corn syrup supplementation were seen as early as two weeks in young adults [20]. Blood samples for laboratory measurements, including plasma lipids and lipoproteins, apolipoproteins B and A1, insulin, high sensitivity (hs) CRP, serum uric acid, alanine aminotransferase (ALT), and aspartate transaminase (AST) were obtained at the end of each 3-week period. During the washout period, participants were not provided any beverages and were allowed to return to their usual consumption of foods and beverages. Participants were required to remain weight-stable for the duration of the study.

Participants visited the Cholesterol Research Center weekly to consult with staff, be weighed, pick up beverages, and fill out questionnaires assessing their beverage intake and diet. They were instructed to drink only their assigned beverages and water, and to maintain stable dietary habits throughout both arms of the study. Intake of non-milk dairy products was allowed in both arms of the study, with the requirement that it remained consistent for the duration of the study. Clinical staff texted participants daily to remind them to consume their assigned beverages. Participants and their parents were asked to consume similar foods for 3 days prior to each blood draw to minimize variation caused by other dietary factors.

This protocol was reviewed and approved by the Institutional Review Board of the University of California, San Francisco Benioff Children’s Hospital Oakland. The trial is registered at clinicaltrials.gov (NCT02094768). Written informed consent was obtained from each participant in accordance with principles of the Declaration of Helsinki.

### 2.2. Participant Recruitment and Enrollment

Participants were recruited from local high schools, internet advertisements and social media postings, advertisements in Children’s Hospital publications, and referrals. Major eligibility criteria included being male, aged 13–18 years, Tanner stage ≥ 2, body mass index (BMI) between 85^th^ and 99^th^ percentile, self-reported intake of 24 oz per day or more of sugar-sweetened soda, no history of diabetes or hypertension, fasting triglyceride <3.39 mmol/L, no nicotine use, and no use of lipid or blood pressure lowering or diabetes medications. Participants were assigned their beverage sequence in randomly determined blocks of 2, 4, 6, and 8 individuals using a uniform random number generator by a statistician. Beverage sequences were kept in sealed envelopes and assigned to the participant by study staff 1–2 days prior to starting the first beverage intervention. Due to the nature of the intervention, participants and clinic staff were not blinded; however, laboratory staff and investigators were blinded until all data had been compiled. Figure 1 displays the details of participant recruitment and enrollment. Thirty participants were randomized and all thirty completed the study.

### 2.3. Diet and Physical Activity Assessment

Participants were asked to complete an online 24-h dietary recall (ASA24-Kids, NCI) [21] weekly to compare dietary intake at baseline (including washout periods), during the milk arm, and during the soda arm. Dietary intake data from completed recalls (ranging from one to three recalls per period) were averaged within each study period. Three participants did not complete any recalls for one or more of the study periods and were thus excluded from the dietary analyses. On average, participants completed 8 ± 1 recalls out of a possible 9 recalls for the entire study. Participants were advised to maintain a constant amount of physical activity throughout the study. They were provided with pedometers to estimate physical activity during the intervention periods. Pedometer counts were collected weekly.

### 2.4. Clinical Procedures

Using standard procedures, blood samples were collected from participants after fasting overnight for 12–14 h. Plasma and serum were separated immediately by centrifugation at 4 °C. Measurements included body weight, height, BMI, BMI *z*-score, and waist circumference measured twice midway between the iliac crest and bottom of the ribs. Body fat percentage was measured on a bioimpedance scale (Tanita TBF-551). Clinical blood pressure measurements were taken after participants remained in a sitting position for at least 5 min. Blood pressure was measured three times, with the last two measurements averaged, and expressed as absolute value and *z*-score.

### 2.5. Laboratory Procedures

Plasma triglycerides, total- and HDL-cholesterol, and glucose were measured by enzymatic endpoint analysis on a clinical chemistry analyzer (LIASYS 330) using methodology previously described [22]. LDL-cholesterol was calculated using the Friedewald equation. Lipid and lipoprotein cholesterol measurements were standardized through the CDC-NHLBI lipid standardization program. Apolipoproteins B and AI were analyzed by immunoturbidimetric assay using the ITA reagent kit. LDL peak particle diameter and plasma concentrations of very low-density lipoprotein (VLDL), intermediate-density lipoprotein (IDL), LDL, and HDL subclasses were analyzed using ion mobility, as previously described [11]. Inter-assay variation was reduced by inclusion of two in-house controls in each preparatory process and duplicate analysis. A coefficient of variation of less than 15% was maintained. Fasting insulin was measured by enzyme-linked immunosorbent assay, with two in-house quality control standards. HOMA-IR was calculated as [glucose (mg/dl) × insulin (mU/L)]/405. Serum uric acid, ALT, and AST were measured using a chemistry analyzer (LIASYS 330) and reagent kits from AMS Diagnostics (Brookfield, CT, USA). Quality control was maintained by the inclusion of two multi chemistry controls (AMS Diagnostics, Brookfield, CT, USA) with each run. hsCRP was measured at a clinical laboratory (Quest Diagnostics).

For sphingolipid analyses, plasma samples (100 μL) were spiked with 10 μL of internal standard mix and subsequently extracted based on protocols described in [23]. Ceramide/Sphingoid Internal Standard Mixture I (Avanti polar lipids; LM6002) was used as an internal standard mix. The detection system was composed of an Agilent 1290 binary gradient ultra-high pressure chromatography system coupled with an Agilent 6490 triple quadrupole mass spectrometer. Sphingolipid metabolites were resolved on a Zorbax RRHD Eclipse Plus C18 column (2.1 × 50 mm; 1.8 micron) fitted with a pre-column composed of identical matrix. Samples were eluted from the column using a binary gradient composed of mobile phase A (2 mM ammonium formate and 0.2% formic acid in 18 mL water) and mobile phase B (1 mM ammonium formate and 0.2% formic acid in MS-grade methanol) at a flow rate of 1 mL/min. Initial composition was 75% B, and this was increased to 80%, 85%, and 100% at 3, 3.1, and 10 min, respectively. The gradient was maintained at 100% B until 8.5 min and subsequently changed back to initial conditions until the end of the run at 10 min. Analysis was carried out using a multiple-reaction-monitoring (MRM) mode. For all compounds, the general source settings in the positive ionization modes were as follows: Gas temperature 200 °C; gas flow, 14 L min^−1^; nebulizer 20 psi; sheath gas temperature 250 °C; sheath gas flow 11 L min^−1^; capillary voltage 3000 V; and nozzle voltage; 0 V. The fragmentor voltage of 380 V and a dwell time of 15 ms were used for all mass transitions, and both Q1 and Q3 resolutions were set to nominal mass unit resolution.

### 2.6. Data Analysis

The primary outcome of the study was LDL peak particle diameter as an index of atherogenic dyslipidemia. Secondary outcomes included other lipid and lipoprotein measurements, measures of adiposity, and blood pressure. Exploratory outcomes included HOMA-IR, hsCRP, serum uric acid, transaminases, and sphingolipids. A sample size of 30 participants in a crossover design was determined to detect a minimum detectable difference of ~1% baseline values for LDL peak particle diameter, based on data from previous measurements in children, at 80% power and α = 0.05 [24]. This would have been sufficient to detect differences previously reported with high sucrose feeding [12]. Paired t-test or Wilcoxon signed-ranked test was used to evaluate comparisons between the soda and milk arms, with 2-tailed *p* < 0.05 considered significant.

## 3. Results

Table 1 shows the characteristics of study participants at the time of screening. BMI and blood pressure means are provided as *z*-scores specific for age and gender groups. On average, the group was overweight, normotensive, and normolipidemic, with the exception of mean HDL-cholesterol being just below the recommended value of 1.17 mmol/L [25]. Physical activity, measured as steps per day, was not different during the two phases of the study (7413 ± 3518 steps per day during the milk phase and 7803 ± 3901 steps per day during the soda phase; *p* = 0.47).

Replacement of soda with milk resulted in decreased sugar, carbohydrate, and caffeine intake, and increased protein intake (Table 2). Total fat intake did not differ between the soda and milk arms whereas saturated fat was significantly higher on the milk arm, attributable to the increased consumption of dairy fat. Cholesterol intake was not different between the soda and milk phases of the study. As would be expected with increased milk consumption, intake of calcium, magnesium, potassium and zinc was also higher during the milk versus soda arm. Although objective measures of compliance were not made, these self-reports of nutrient intake, based on weekly dietary recalls, suggest good compliance in consuming the test beverages. Dietary compliance scores were 4.8 ± 0.4 out of 5 for the soda intervention and 4.6 ± 0.6 for the milk intervention.

Table 3 shows the effects of replacing soda with milk on anthropomorphic, biochemical, and metabolic outcomes. As intended, weight stayed constant throughout the study, as did waist circumference. Replacing 24 oz. of soda with reduced fat milk did not affect LDL peak particle diameter nor any other measured lipid and lipoprotein variable, i.e., triglycerides, total-, LDL-, or HDL-cholesterol, apolipoprotein B, apolipoprotein AI, and lipoprotein subfractions. HOMA-IR, hsCRP, and liver enzymes were also unaffected by the intervention. In contrast, systolic blood pressure and uric acid concentrations were significantly reduced after the milk intake phase compared to the soda intake phase. Replacement of soda with milk led to decreases in systolic blood pressure in two-thirds of individuals. Uric acid concentrations correlated positively with systolic blood pressure (*r* = 0.43; *p*= 0.02) following the soda intake phase, but not the milk intake phase.

**Figure 2:**
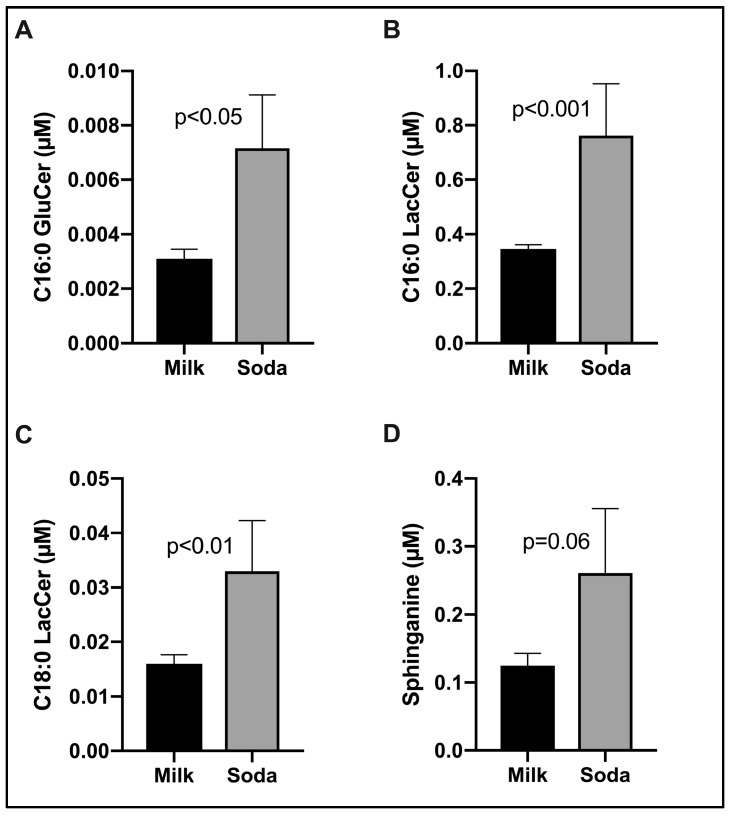
Replacement of SSB intake with milk significantly decreased glycosphingolipids in 30 healthy overweight/obese adolescent boys. Figures show mean ± SEM of plasma glucosylceramide (GluCer, Panel **A**), lactosylceramide (LacCer, Panel **B**, **C**) and sphinganine (Panel **D**). *p* values were calculated based on Wilcoxon signed rank test.

Figure 2 shows the effects of soda replacement with milk on plasma SPLs. Our panel consisted of broad coverage SBs, phosphorylated SBs, Cers, phosphorylated Cers, mono- and di-hexosylCers, and SMs. Of these, the transition from SSB to milk was associated with a significant reduction in C16:0 glucosylceramide (GluCer; panel A, Figure 2; *p* < 0.05). Correspondingly, C16:0 and C18:0 lactosylceramides (LacCer), that are synthesized from GluCer, also decreased following soda replacement with milk (Figure 2B,C). In addition, plasma sphinganine (Spa), a key de novo substrate for Cer synthesis, also trended lower following milk replacement (Figure 2D). No other differences in SPL metabolites were observed. Collectively, the data suggest that plasma HexCers may be the most sensitive metabolic biomarkers of decreased SSB intake and/or increased milk intake in young normolipidemic adolescents.

## 4. Discussion

Recent public health efforts have focused on reducing consumption of sugar-sweetened beverages in children and adolescents due to strong evidence of their deleterious effects on health. We sought to determine whether replacement of sugar-sweetened soda with reduced fat milk would improve markers of cardiometabolic health in adolescent boys. We chose to study adolescent males because, as a group, they consume the greatest amounts of sugar-sweetened beverages [26]. Furthermore, there are documented gender differences in insulin sensitivity and plasma lipid changes that occur during adolescence [27], with adolescent boys possessing a greater likelihood of expressing features of atherogenic dyslipidemia than girls [28]. We hypothesized that in habitual soda drinkers, the replacement of 24 oz of soda containing ~80 g high fructose corn syrup with an energy equivalent amount of reduced fat milk would increase LDL peak particle diameter. Smaller LDL particle diameter is a marker for atherogenic dyslipidemia [29] and is known to be modifiable by diet. However, in this short term, randomized dietary crossover trial, we did not observe a significant effect of replacing soda with milk on LDL particle diameter. Other biomarkers of cardiometabolic health, including triglycerides, total cholesterol, LDL-cholesterol, HDL-cholesterol, apolipoproteins B and AI, and lipoprotein subfraction concentrations, HOMA-IR as an indicator of glucose homeostasis, hsCRP as a marker for inflammation, and liver enzymes, were also unaffected by the intervention. However, systolic blood pressure and uric acid concentrations were significantly reduced with replacement of soda by milk.

The lack of change in LDL peak particle diameter with milk versus soda consumption in this study contrasts with previous data documenting decreases in LDL particle size or greater abundance of small, dense LDL in weight-stable individuals consuming low fat, high carbohydrate diets [18,24] and 80 g/day fructose or 80 g/day sucrose feeding [9]. The effects of milk or dairy consumption on LDL particle size are less clear, with studies showing neutral, positive, or adverse effects, and the type of dairy food and fat content modulating their effects [30]. As expected, LDL peak particle diameter in our study population was inversely and significantly correlated with plasma triglyceride and waist circumference as a measure of visceral adiposity (data not shown). However, it is notable that the mean LDL peak particle diameter after both the soda and milk intervention periods was relatively large, as measured by ion mobility, i.e., 22.4 nm, a size categorized as pattern A, which is characterized by a greater abundance of larger and more buoyant lipoprotein particles and associated with lower CVD risk than a predominance of smaller LDL (pattern B) [11,31]. There were only two individuals per treatment arm who exhibited pattern B, a prevalence of ~6%. While this is comparable to what we have previously reported in normal weight children and adolescents [32], even following consumption of a very high carbohydrate diet [24], this finding was unexpected given that study participants were overweight and obese. Two studies have reported a high prevalence (40% to 55%) of pattern B in overweight and obese children [33,34]. Thus, the teens in our study appeared to be protected from the adverse lipid profile that commonly accompanies overweight. The basis for the observed normolipidemia in our overweight and obese study cohort is not known, but it is possible that the teens in this study, who were not insulin resistant and had normal hsCRP levels, were metabolically fit in spite of being overweight [35]. Their average body fat percentage was in the range considered as ‘high adiposity’ (>75th percentile for age and sex) [36,37] and their physical activity, as measured by a pedometer, was not greater than in previous studies of normal and obese adolescents [38,39]. Their ability to maintain normal lipid levels may be related to intrinsic metabolic machinery that also allows them to efficiently burn fuel, including the sugar consumed from sodas. Previous studies in children and adolescents that showed significant lipid improvements with dietary fructose or sugar restriction focused on individuals with metabolic syndrome [40] and documented nonalcoholic fatty liver disease [41,42], suggesting that these effects may only be apparent in populations with higher cardiometabolic risk. Furthermore, a recent meta-analysis suggested that effects of high fructose consumption on lipid levels are only apparent in hypercaloric, and not isocaloric, conditions [43]. By design, the study participants maintained their weight during the study, which may have contributed to the lack of observed differences. We do not believe that our methods of recruitment were biased towards obese, but healthy, adolescents, although it is possible that there may have been unintentional selection for these individuals.

Despite the lack of significant effects on plasma lipoproteins of changing from SSB to milk, significant decreases in glycosphingolipids (LacCer and GluCer) were observed (Figure 2). GluCer and LacCer are the two most abundant glycosphingolipids and are mostly (~60%) distributed in LDL particles [44,45]. Pre-clinical studies have shown beneficial effects of glycosphingolipid synthesis in improving insulin sensitivity [46] and atherosclerosis [47]. LacCer has also been found to be positively correlated with arterial stiffness among middle-aged men [48]. Increased GluCer has been shown to impair insulin sensitivity in adipocytes [49]. These results suggest that favorable shifts in LDL SPL composition may occur even in the absence of significant changes in levels of LDL particles.

As mentioned above, systolic blood pressure and uric acid were significantly reduced when milk was consumed in place of soda. These beneficial effects are consistent with results of previous studies that have shown similar responses in adults [10,50]. The metabolism of fructose has been reported to increase uric acid production, and serum uric acid levels have been shown to increase with sugar consumption [20,51,52]. The effects of increased fructose intake on blood pressure may be mediated at least in part by uric acid. Uric acid impairs endothelial function and vascular compliance [51], and epidemiological studies have reported an approximately 1.5- to 2-fold increased risk of hypertension with increased uric acid concentrations [51]. In children, a strong linear association between serum uric acid concentrations and systolic blood pressure has been observed [53]. Furthermore, a previous cohort study in adolescents reported associations between higher SSB intake and increases in serum uric acid levels and systolic blood pressure [54]. Studies in humans and animal models have demonstrated fructose-induced increases in uric acid levels and blood pressure [55,56]. Two recent intervention studies in children with metabolic syndrome [40] and nonalcoholic fatty liver disease [41] reported reduced diastolic and systolic blood pressure, respectively, with reduced fructose consumption. However, the effects on blood pressure in these studies may also have been mediated by reductions in body weight [40] and body fat [41] that occurred concomitantly.

Improvements in blood pressure with milk replacement of soda may also have been due to the beneficial effects of milk consumption. Epidemiological evidence has shown a favorable association of dairy product consumption with blood pressure [57,58]. A dose-response meta-analysis of prospective cohort studies found a 4% decrease in the incidence of hypertension for every 200 g/day of milk intake (our study provided 671 g/day), while high-fat dairy, total fermented dairy, yogurt, and cheese were not associated with hypertension incidence [57]. The results of clinical trials have been inconsistent, with a recent meta-analysis of randomized trials showing no effect of dairy on systolic blood pressure in interventions lasting one month to one year [59]; however, the analyses did not consider the source or type of dairy. In one recent trial, the known blood pressure lowering effects of the Dietary Approaches to Stop Hypertension (DASH) diet were shown to be independent of the saturated fatty acid content (provided as dairy) of the diets [22]. Beneficial effects of dairy on blood pressure may be due to milk proteins or other nutrients contained in dairy products, such as potassium, calcium, and magnesium, or bioactive peptides. Although there is limited evidence to support such effects, a meta-analysis of clinical trials showed a small but significant effect of milk proteins on reducing blood pressure [60].

There are several limitations to the present study including the short intervention period, restriction to males, and reliance on self-reports to evaluate dietary compliance. In addition, assessment of habitual physical activity by pedometer does not account for the intensity of activity or for calories burned for a given level of exertion. Given the apparent metabolic fitness of our study participants, it would be worthwhile in future studies to investigate the effects of milk vs. SSBs employing more comprehensive evaluations of energy metabolism, for example, through indirect calorimetry and exercise-challenged respiratory rates.

## 5. Conclusions

Overweight and obese normolipidemic adolescent males who were habitual soda drinkers did not show further improvement in lipid and lipoprotein measurements in response to short-term isocaloric replacement of sugar-sweetened soda with reduced fat milk. Coordinated reductions in systolic blood pressure and serum uric acid concentrations, as well as changes in glycosphingolipids, suggest potential cardiometabolic benefits of this intervention, but these results require confirmation in future studies.

## Figures and Tables

**Table 1:** Participant screening characteristics (*n* = 30).

	Mean ± SD
Age, y	15.3 ± 1.5
Weight, kg	86.8 ± 15.7
BMI, *z*-score	1.8 ± 0.5
Body fat, %	29 ± 9
Systolic BP, mmHg	119 ± 9
Systolic BP, *z*-score	0.2 ± 0.8
Diastolic BP, mmHg	60 ± 6
Diastolic BP, *z*-score	−0.5 ± 0.6
Triglyceride, mmol/L	1.02 ± 0.48
Total cholesterol, mmol/L	3.75 ± 0.50
LDL-cholesterol, mmol/L	2.14 ± 0.39
HDL-cholesterol, mmol/L	1.15 ± 0.26
Glucose, mmol/L	5.64 ± 0.28

Abbreviations: BP, blood pressure; LDL, low-density lipoprotein; HDL, high-density lipoprotein.

**Table 2:** Nutrient intake assessed by 24-h recall surveys ^1,2^.

	Baseline	Soda	Milk	*p*-Value ^3^ (milk vs. soda)
Carbohydrate, %E	48.8 ± 2.1	47.2 ± 1.4	41.5 ± 1.4	0.0002
Sugar, %E	23 ± 2	22± 1	16 ± 1	<0.0001
Total fat, %E	36.1 ± 1.4	37.4 ± 1.3	38.5 ± 1.0	0.35
SFA, %E	12.7 ± 0.5	13.0 ± 0.6	14.8 ± 0.5	0.004
MUFA, %E	13.1 ± 0.6	14.2 ± 0.6	13.7 ± 0.5	0.53
PUFA, %E	7.1 ± 0.5	7.9 ± 3.2	6.7 ± 0.4	0.02
Protein, %E	16.1 ± 0.7	16.0 ± 0.7	20.1 ± 0.8	<0.0001
Calcium, mg	995 ± 115	716 ± 58	1389 ± 108	<0.0001
Cholesterol, mg	281 ± 30	275 ± 35	328 ± 30	0.13
Magnesium, mg	252 ± 29	199 ± 15	261 ± 18	0.0002
Potassium, mg	2225 ± 214	1680 ± 161	2392 ± 153	<0.0001
Zinc, mg	12.6 ± 1.1	10.6 ± 0.7	14.0 ± 1.0	0.002
Caffeine, mg	23.7 ± 5.6	21.4 ± 4.2	4.1 ± 1.6	0.0002

^1^*n* = 27. Three participants did not complete at least one survey per diet period and were excluded from analyses. ^2^ Values are means ± SE and represent the average of the completed dietary recalls per diet period (one to three recalls for each of the three diet periods). The average total number of completed surveys per participant was 8 ± 1 (out of maximum of 9). Abbreviations: %E, % energy; SFA, saturated fatty acid; MUFA, monounsaturated fatty acid; PUFA, polyunsaturated fatty acid. ^3^ Significance was determined by paired t-test or Wilcoxon signed rank test for non-normally distributed variables.

**Table 3:** Matched pair analyses of the effects of soda or milk on study outcomes ^1^.

	Soda	Milk	*p*-Value ^2^
Weight, kg	87.7 ± 16.1	87.7 ± 16.2	0.43
Waist circumference, cm	86 ± 10	88 ± 12	0.32
Body fat, %	29 ± 10	30 ± 10	0.27
LDL peak particle diameter, nm	22.4 ± 0.6	22.4 ± 0.5	0.73
Systolic BP, mmHg	118 ± 10	116 ± 7	0.06
Systolic BP, *z*-score	0.2 ± 1.0	0.0 ± 0.8	0.04
Diastolic BP, mmHg	62 ± 6	62 ± 4	0.82
Diastolic BP, *z*-score	−0.4 ± 0.5	−0.4 ± 0.4	0.69
Triglyceride, mmol/L	0.89 ± 0.49	1.05 ± 0.66	0.09
Total cholesterol, mmol/L	3.56 ± 0.52	3.60 ± 0.55	0.53
LDL-cholesterol, mmol/L	2.04 ± 0.41	2.02 ± 0.43	0.63
HDL-cholesterol, mmol/L	1.10 ± 0.27	1.10 ± 0.25	0.84
Apolipoprotein B, g/dL	0.60 ± 0.13	0.61 ± 0.15	0.48
Apolipoprotein A1, g/dL	1.16 ± 0.21	1.17 ± 0.20	0.57
Lipoprotein particle concentrations, nmol/L			
Large VLDL	12.2 ± 8.3	15.2 ± 11.1	0.14
Medium VLDL	33.8 ± 18	37.7 ± 20.3	0.25
Small VLDL	31.6 ± 10.3	32 ± 11.2	0.84
IDL	82.9 ± 21.3	85.5 ± 25	0.76
Large LDL	490 ± 142	501 ± 132	0.61
Med LDL	134 ± 63	134 ± 63	0.09
Small LDL	89 ± 41	101± 59	0.21
Very small LDL	143 ± 48	136 ± 37	0.26
Small HDL	12,930 ± 2176	13,434 ± 2522	0.15
Large HDL	5481 ± 1078	5596 ± 1200	0.48
HOMA-IR	0.54 ± 0.21	0.54 ± 0.23	0.96
Uric acid, μmol/L	381 ± 58	362 ± 62	0.02
hsCRP, nmol/L	11.6 ± 13.4	10.6 ± 11.1	0.75
ALT, U/L	27.2 ± 7.7	28.4 ± 8.3	0.39
AST, U/L	20.9 ± 5.2	22.4 ± 4.9	0.09

^1^ Values are mean ± SD, *n* = 30. ^2^ Significance was determined by paired t-test or Wilcoxon signed rank test for non-normally distributed variables. Abbreviations: ALT, alanine aminotransferase; AST, aspartate transaminase; HDL, high-density lipoprotein; HOMA-IR, homeostatic model assessment of insulin resistance; hsCRP, high sensitivity C-reactive protein; IDL, intermediate-density lipoprotein; LDL, low-density lipoprotein; VLDL, very low-density lipoprotein.
